# Establishment of a Humanized APL Model via the Transplantation of *PML-RARA*-Transduced Human Common Myeloid Progenitors into Immunodeficient Mice

**DOI:** 10.1371/journal.pone.0111082

**Published:** 2014-11-04

**Authors:** Hiromichi Matsushita, Takashi Yahata, Yin Sheng, Yoshihiko Nakamura, Yukari Muguruma, Hideyuki Matsuzawa, Masayuki Tanaka, Hideki Hayashi, Tadayuki Sato, Anar Damdinsuren, Makoto Onizuka, Mamoru Ito, Hayato Miyachi, Pier Paolo Pandolfi, Kiyoshi Ando

**Affiliations:** 1 Research Center for Cancer Stem Cell, Tokai University School of Medicine, Isehara, Kanagawa, Japan; 2 Medical Research Institute, Tokai University, Isehara, Kanagawa, Japan; 3 Department of Laboratory Medicine, Tokai University School of Medicine, Isehara, Kanagawa, Japan; 4 Department of Cell Transplantation, Tokai University School of Medicine, Isehara, Kanagawa, Japan; 5 Support Center for Medical Research and Education, Tokai University, Isehara, Kanagawa, Japan; 6 Division of Hematology and Oncology, Department of Internal Medicine, Tokai University School of Medicine, Isehara, Kanagawa, Japan; 7 Central Institute for Experimental Animals, Kawasaki, Kanagawa, Japan; 8 Cancer Research Institute, Beth Israel Deaconess Cancer Center, Departments of Medicine and Pathology, Beth Israel Deaconess Medical Center, Harvard Medical School, Boston, Massachusetts, United States of America; University of Texas M.D. Anderson Cancer Center, United States of America

## Abstract

Recent advances in cancer biology have revealed that many malignancies possess a hierarchal system, and leukemic stem cells (LSC) or leukemia-initiating cells (LIC) appear to be obligatory for disease progression. Acute promyelocytic leukemia (APL), a subtype of acute myeloid leukemia characterized by the formation of a PML-RARα fusion protein, leads to the accumulation of abnormal promyelocytes. In order to understand the precise mechanisms involved in human APL leukemogenesis, we established a humanized *in vivo* APL model involving retroviral transduction of *PML-RARA* into CD34^+^ hematopoietic cells from human cord blood and transplantation of these cells into immunodeficient mice. The leukemia well recapitulated human APL, consisting of leukemic cells with abundant azurophilic abnormal granules in the cytoplasm, which expressed CD13, CD33 and CD117, but not HLA-DR and CD34, were clustered in the same category as human APL samples in the gene expression analysis, and demonstrated sensitivity to ATRA. As seen in human APL, the induced APL cells showed a low transplantation efficiency in the secondary recipients, which was also exhibited in the transplantations that were carried out using the sorted CD34^−^ fraction. In order to analyze the mechanisms underlying APL initiation and development, fractionated human cord blood was transduced with *PML-RARA*. Common myeloid progenitors (CMP) from CD34^+^/CD38^+^ cells developed APL. These findings demonstrate that CMP are a target fraction for *PML-RARA* in APL, whereas the resultant CD34^−^ APL cells may share the ability to maintain the tumor.

## Introduction

Acute myeloid leukemia (AML) constitutes a heterogeneous group of tumors in myeloid lineage cells characterized by the proliferation and accumulation of immature myeloblasts [1]. Recent advances in cancer biology have revealed that various genetic events result in the blockage of differentiation with subsequent uncontrolled cellular proliferation. In addition, *in vivo* analyses using a xenograft model with immunodeficient mice have shown that a very immature subset of AML cells called leukemic stem cells (LSC), which are typically characterized as CD34^+^/CD38^−^ cells, as observed in normal hematopoietic stem cells (HSCs), have been shown to slowly undergo cell division to both yield progenitor cells and sustain the LSC population, thus resulting in the maintenance of the tumor [2]–[6]. More recently, several reports have shown that CD34^+^/CD38^+^ hematopoietic progenitors are able to acquire the ability to maintain populations of LSC or leukemia-initiating cells (LIC) [7]. It is therefore possible that the phenotypes of LIC differ among the subtypes of AML.

Acute promyelocytic leukemia (APL) is a subset of AML defined by the formation of a chimeric gene, promyelocytic leukemia-retinoic acid receptor α (*PML-RARA*) [8]. It is characterized by the accumulation of abnormal promyelocytes with abundant large azurophilic granules, suggesting that APL cells undergo maturation arrest in the later steps of myeloid differentiation. The typical pattern of cellular surface markers of APL is positive for CD13, CD33 and CD117, and negative for CD34, which is usually presumed to indicate cellular immaturity, and HLA-DR [9]. It is very difficult to engraft primary APL samples in immunodeficient mice. They did not become engrafted into the NOD/SCID mice to any degree [3]. In NOD/Shi-SCID/IL-2Rγ^null^ (NOG) mice, which are more profoundly immunocompromised than NOD/SCID mice [10], [11], six out of eight APL samples were not engrafted or only very little engrafted [12]. It is therefore possible that the mechanisms underlying the development of APL differ from those involved in the pathogenesis of AML uncovered to date. Elucidating the pathogenesis of APL is important for improving the treatment of APL patients, and will provide clues to understand the development of other subtypes of AML.

*In vivo* analyses using transgenic APL mice models with *PML-RARA* have revealed that a population of committed myeloid progenitor cells (CD34^+^, c-kit^+^, FcγRIII/II^+^, Gr1^int^) was identified as the APL-LIC [13], [14]. However, the cellular surface antigens and the gene expression pattern in humans are different from those in mice. In particularly, in transgenic systems, murine APL developed after a long latent period through a myelodysplastic/proliferative phase, which does not usually precede human APL [15]–[18]. There have been no *in vivo* models for exploring leukemogenesis of human APL to date; largely because human primary APL cells are difficult to engraft as a xenograft [3], [12]. *PML-RARA*-retrovirally transduced human CD34^+^ cells from cord blood have therefore only been evaluated *in vitro* [19].

Therefore, the aim of this study was to establish a humanized xenograft APL model using the retroviral transduction of *PML-RARA* into human CD34^+^ cells and NOG mice in order to investigate the mechanisms of APL leukemogenesis, such as that involving disease initiation and maintenance in the model.

## Materials and Methods

### Fractionation of human hematopoietic cells from cord blood

Cord blood (CB) and patients' APL samples were obtained after written informed consent was provided in accordance with the Declaration of Helsinki and with approval from the Tokai University Committee on Clinical Investigation (Permit number: #12I-46 and #12I-49). CD34 positive and negative specimens were primarily prepared using the CD34 Progenitor Cell Isolation Kit (Miltenyi Biotec, Bergisch Gladbach, Germany). CD34^+^ cells were then purified again using anti-human CD34 mAbs (Beckman Coulter, Brea, CA), in combination with or without an anti-CD38 antibody (BD, Franklin Lakes, NJ), with a FACS vantage instrument (BD). CD34^−^/CD33^+^ cells were also purified again using anti-human CD34 and CD33 mAbs (Beckman Coulter) and the FACS vantage instrument. The preparation of common myeloid progenitors (CMP), granulocyte-monocytic progenitors (GMP) and megakaryocyte-erythrocyte progenitors (MEP) was performed using an anti-CD123 antibody (BD) and anti-CD45RA (Biolegend, San Diego, CA) antibody, according to a previous report [20].

### Retrovirus transduction of *PML-RARA* into human hematopoietic cells

The MIGR1 retroviral vector [21] or MIGR1-*PML-RARA* (bcr3/short form) [22] in combination with the vesicular stomatitis virus-G protein (VSV-G) envelope vector (pCMV-VSV-G) was transiently transfected into PLAT-gp cells using the Fugene 6 transfection reagent (Roche Diagnostics, Basel, Switzerland). The culture supernatant was concentrated 100 to 200 times by ultracentrifugation. After overnight culture of the fractionated cells in StemPro-34 (Life Technologies, Carlsbad, CA) with TPO, SCF, and FLT3 ligand (50 ng/ml each), they were incubated with the concentrated supernatant on retronectin-coated plates (Takara-Bio, Otsu, Japan). Retroviral transduction was performed twice, and then transplantation was performed the next day.

### Colony-forming unit-cells assay

*PML-RARA* transduced cells were sorted by their EGFP, CD34 and CD38 expression by FACS vantage 48 h after infection. The colony-forming unit-cells (CFU-C) assay was performed as described previously [23]. The fluorescent images were captured using a HS All-in-One Fluorescence Microscope Biorevo 9000 (Keyence Corporation, Osaka, Japan) and were analyzed by the BZ II software program (Keyence Corporation).

### RNA extraction and RT-PCR

Total RNA was isolated using the RNeasy micro kit (Qiagen, Hilden, Germany) or Isogen (Nippon gene, Tokyo, Japan), and the reverse-transcribed cDNA was amplified by qualitative PCR. The qualitative-PCR analysis was performed by SRL Inc. (Hachioji, Tokyo, Japan). The sequences of PCR primers and probes were shown in **Table S1**.

### Transplantation, serial transplantation and ATRA treatment

Nine- to 20-week-old NOD/Shi-scid, IL-2Rγc^null^ (NOG) mice [10], [11] were irradiated with 220 cGy of X-rays. On the following day, the whole infected cells or primary AML cells were intravenously injected. The EGFP-positive cells in the peripheral blood were monitored. The mice with induced APL were defined as those bearing more than 0.1% EGFP^+^ cells which dominantly expressed CD33 (more than 70%) in their bone marrow at four months after transplantation. In the initial analysis, the occurrence of APL was confirmed by the morphological observations using cytospin slides after EGFP sorting. For serial transplantation, bone marrow cells were obtained from recipient mice, and the sorted EGFP-positive cells were injected intravenously or intramedullary into the irradiated mice [24]. The engrafted mice were treated intraperitoneally with 1.5 µg/g of body weight/day of all-*trans* retinoic acid (ATRA, Sigma) for 21 days [25], and were then sacrificed to collect the EGFP-positive cells in the bone marrow by sorting. All the experiments using animals were approved by the animal care committee of Tokai University (Permit number: #132028).

### Flow cytometric analysis

The cells were stained with APC-conjugated anti-human CD45, CD33, CD34, HLA-DR (Beckman Coulter), CD13, and CD117 (BD) mAbs. They were subjected to flow cytometry using a FACSCalibur instrument (BD) and the CellQuest software program (BD).

### Cell preparation, Wright-Giemsa staining and immunofluorescence microscopy

Cytospin slides were prepared using a Cytospin 4 Cytocentrifuge (Thermo Scientific, Waltham, MA) at 500 rounds per minute for 5 min. To observe the cellular morphology, Wright-Giemsa staining was performed. For the immunofluorescent study, cells were seeded onto poly-L-lysine coated slides and fixed with ice cold 70% ethanol for 15 min. After permeabilization with 0.2% Triton X-100 for 20 min, the slides were treated with PBS containing 5% normal goat serum for 1 hour to block the nonspecific binding of antibodies. The anti-PML antibody (Merck Millipore, Billerica, MA) was applied overnight at 4°C. Cells were counterstained with DAPI. Images were captured with an LSM510 META confocal microscope (Carl Zeiss, Oberkochen, Germany) and processed using Adobe Photoshop 7.0 (Adobe Systems, San Jose, CA).

### Southern blot analysis

Genomic DNA was extracted from the cells using a DNeasy kit (Qiagen). Ten micrograms of the DNA were electrophoresed and transferred to nylon membranes (Hybond-N+, GE Healthcare, Fairfield, CT). The DNA was then crosslinked to the membrane by ultraviolet light. The EGFP probe was prepared from MIGR1 vector by cutting it using NcoI and SalI, and was labeled with ^32^P-dCTP using the Rediprime II DNA Labelling System (GE Healthcare). The membrane was hybridized with the probe in Rapid-hyb buffer (GE Healthcare), and was analyzed by a Phosphoimager (LAS1000, Fuji Film, Tokyo, Japan).

### Microarray

Total RNA was labeled and hybridized to Affymetrix Human Genome U133 Plus 2.0 Array GeneChip microarrays (Affymetrix, Santa Clara, CA) using the manufacturer's protocols. The results were deposited in the Gene Expression Omnibus (GEO; http://www.ncbi.nlm.nih.gov/geo/; accession no. GSE49344). The microarray data from normal human promyelocytes and clinical samples bearing AML, including APL, were obtained from the deposited data on the GEO (GSE12662). The APL-specific expressed genes have been described in detail in a previous study [26]. The probe set data were generated using standard normalization algorithms included in the Affymetrix Microarray Suite software program, v.5 (MAS5.0). The clustering analysis was performed by the Gene spring GX software program, version 11 (Agilent technology, Santa Clara, CA).

To identify the genes that are differentially expressed in a specific cellular subset, all probe sets with fewer than 10% present calls in both groups and a coefficient of variation <0.5 across all samples were eliminated prior to the subsequent analysis; (i) The genes differently expressed in the induced APL cases among the AML cases were defined as genes whose expression change (upregulation or downregulation) was ≥2.0 fold in comparison to those in AML other than APL, (ii) The genes associated with immaturity in the induced APL cases were expressed at significantly higher levels in CD34^+^ cells than the other normal cells (FDR <0.05, fold-change >2.0 upregulated), and were also expressed in the induced APL cells at similar levels (induced APL: CD34^+^ ≥1∶1), and moreover, these genes were expressed at higher levels in promyelocytes (induced APL: Pros ≥2∶1). (iii) The genes upregulated during promyelocyte differentiation were expressed at significantly higher levels in promyelocytes than in the other normal cells (FDR <0.05, fold-change >2.0 upregulated), and were expressed at lower levels in the induced APL (induced APL: Pros ≤1∶2), (iv) The genes induced by *PML-RARA* were expressed at higher levels in the induced APL than in all the normal cells (induced APL: normal ≥2∶1), and were not expressed in any normal cells (more than 75% absent calls, as summarized by the MAS5.0). Statistical significance was assessed by the unpaired unequal variance Welch test (P<0.05), and correction for multiple testing was performed by the Benjamini and Hochberg False Discovery Rate (FDR), using a cutoff of 0.05.

The gene set specific for induced APL was defined to fulfill one or more of the above criteria (ii) to (iv) in the gene set identified in (i).

### The integration site analysis of *PML-RARA* using linear amplification-mediated PCR

To identify the genomic-proviral junction sequence, linear amplification-mediated polymerase chain reaction (LAM-PCR) was performed as described previously, with minor modifications [27], [28]. In brief, genomic DNA from bone marrow cells was first digested with Tsp509I. A linear amplification of target DNA in the digested genome was performed by repeated primer extension using a vector-specific 5′-biotinylated primer, LTR1.5, and Taq polymerase. After selection with Dynabeads MyOne Streptavidin C1 (Life Technologies), a double-stranded asymmetrical linker cassette was ligated to the Tsp509I-digested site using T4 DNA Ligase. The DNA products were then amplified by PCR using a vector-specific primer, LTR3, and linker cassette primer, LC1. The nested PCR was performed using internal primers LTR5 and LC2. The final products were sequenced after cloning them into the TOPO TA cloning vector (Life Technologies). The primer sequences are shown in **Table S1**.

### Statistical analyses

The Kaplan-Meier method was used to estimate the leukemia-free survival (LFS) of mice. Log-rank p values were used for comparisons of the LFS among three subgroups. The analyses were conducted using the GraphPad Prism software package (GraphPad Software, La Jolla, CA). The other statistical analyses were performed using the Mann-Whitney U Test with the IBM SPSS Statistics software program (New York, NY). Values of p<0.05 were considered to be statistically significant.

## Results

### Functions of PML-RARα in human CD34 cells *in vitro*

**Figure 1:**
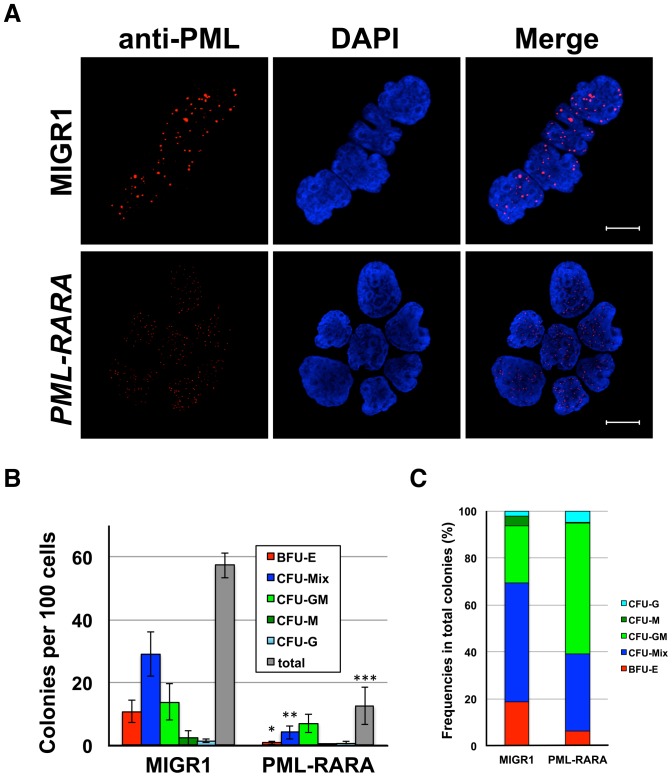
The function of *PML-RARA* in human CD34^+^ cells *in vitro*. (**A**) The results of an immunofluorescent analysis of PML distribution in human CD34^+^ cells transduced with *PML-RARA*. The images were captured with an LSM510 META confocal microscope (Carl Zeiss). The bars indicate 10 µm. (**B**) The colony-forming assay using *PML-RARA*-transduced CD34^+^ cells. The cells were sorted by EGFP expression 48 h after infection. Colony formation was evaluated on days 10 to 12 after plating the cells. The average numbers of colonies from three independent experiments are shown. Data represent the means ± SD. The asterisks (*) indicate p<0.05. (**C**) The proportion of each kind of colony was calculated from the results of the colony-forming assay shown in (B). The percentages of CFU-GM are higher in *PML-RARA*-infected cells than in control (MIGR1) cells (p = 0.013).

To examine the functions of PML-RARα *in vitro*, a MIGR1-*PML-RARA* or MIGR1 control vector was retrovirally infected into human CD34^+^ cells from cord blood. The transduction efficiency evaluated by EGFP positivity was 0.5% to 18.6% (n = 10, median: 5.7%) and 2.1% to 19.7% (n = 25, median: 6.8%), respectively. The expression of *PML-RARA* in these cells was confirmed by RT-PCR (**Figure S1**). The induction of *PML-RARA* in CD34^+^ cells disrupted PML nuclear bodies by interacting with wild-type PML via the PML portion of the chimeric transcript, and the distribution of PML in the nucleus was altered to show a microspeckled pattern in these cells [29]–[31] (**Figure 1A**). Additionally, the induction of *PML-RARA* reduced all the colony formation capacity (**Figure 1B**). The *PML-RARA* expression was confirmed in these colonies by EGFP fluorescence (**Figure S1 and S2**) and RT-PCR, although the expression levels were 37 times lower compared to those in the CD34^+^ cells 48 hours after *PML-RARA* transduction (**Figure S3**). Regarding the content of colonies, MIGR1-infected CD34^+^ cells mainly generated the erythroid lineage-containing colonies, such as CFU-mix and BFU-E, whereas more than half of the colonies from the CD34^+^ cells with *PML-RARA* were of the myeloid lineage, like CFU-GM (**Figures 1C**). These data demonstrate that *PML-RARA* induces the myeloid commitment of human CD34^+^ cells.

### Establishment of a humanized *in vivo* APL model

**Figure 2:**
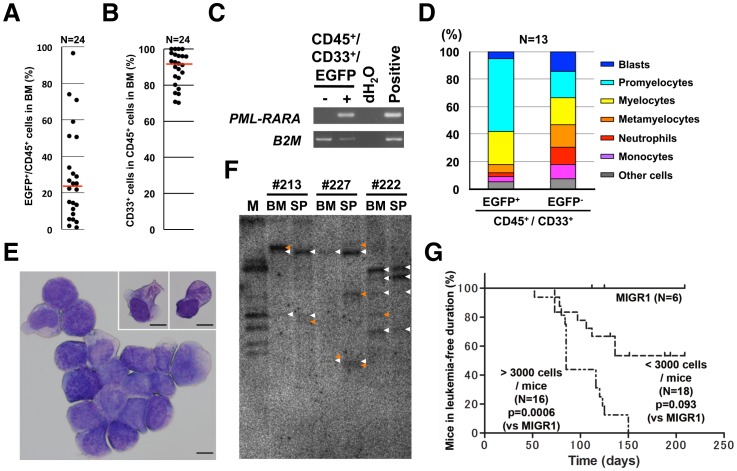
Establishment of humanized APL *in vivo*. (**A**) The proportion of EGFP^+^/human CD45^+^ cells in the bone marrow of leukemic NOG mice. Each dot represents a single mouse. The horizontal line represents the median value. (**B**) The proportion of CD33^+^ cells among the EGFP^+^/human CD45^+^ cells in the bone marrow of the leukemic NOG mice. Each dot represents a single mouse. The horizontal line represents the median value. (**C**) The expression of *PML-RARA* in RT-PCR was detected only in the EGFP^+^ fraction obtained from the engrafted human CD45^+^/CD33^+^ cells. The cells were obtained from bone marrow 16 weeks after transplantation. *B2M, beta 2 microglobulin*. The *PML-RARA* expression vector and human CD34^+^ cells were used as a positive control for the *PML-RARA* and *B2M* analysis, respectively. (**D**) The differential counts of the engrafted CD45^+^/CD33^+^/EGFP^+^ and EGFP^−^ cells from the mice transplanted with *PML-RARA*-induced human CD34^+^ cells. They were obtained from bone marrow 16 to 20 weeks after transplantation. The data represent the means. (**E**) A representative photograph of the resulting leukemic cells which morphologically recapitulated APL. Faggot cells were recognized, as seen in the right top corner. The images were captured with a BX41 microscope (Olympus). The bar indicates 10 µm. (**F**) The results of a Southern blot analysis of the genomic DNA from induced APL cells with an EGFP probe. Clonal bands are shown by arrow heads: white, seen in both BM and SP; orange, seen only in BM or SP. BM, bone marrow; SP, spleen. (**G**) The leukemia-free duration in NOG mice transplanted with *PML-RARA-*transduced CD34^+^ cells.

The cells transduced with *PML-RARA* or the MIGR1 control vector were then transplanted into NOG mice. The EGFP^+^ cells survived and proliferated three to four months after transplantation only in the NOG mice transplanted with *PML-RARA-*, not control vector at all, infected human CD34^+^ cells. The median proportion of EGFP^+^/CD45^+^ cells in the bone marrow obtained from the transplanted mice was 23.7% (0.95% to 96.5%, n = 24) (**Figure 2A**). The majority of the engrafted EGFP^+^/human CD45^+^ cells expressed human CD33 (70.2% to 100%, median 90.6%, n = 24) (**Figure 2B**), thus suggesting that they were of the myeloid lineage. The *PML-RARA* expression was detected only in the EGFP^+^ fraction and not in the EGFP^−^ fraction of the sorted human CD45^+^/CD33^+^ cells from the NOG mice (**Figure 2C**). The expression levels of *PML-RARA* were decreased about 17-fold in comparison to those in the CD34^+^ cells 48 hours after *PML-RARA* transduction (**Figure S3**), but the presence of P*ML-RARA* in the human myeloid cells, recognized as EGFP^+^ cells, caused marked accumulation of promyelocytes, in comparison to the control EGFP^−^ human myeloid cells (52.8% in EGFP^+^ cells vs 19.4% EGFP^−^ cells in the 13 paired samples, p<0.0001). On the other hand, the proportions of myeloblasts, mature neutrophils and monocytes were decreased (5.2% vs 14.4%, p = 0.010; 2.5% vs 12.7%, p = 0.005; 4.0% vs 10.1%, p = 0.016) (**Figure 2D**). These findings confirmed that the expression of *PML-RARA* induced the myeloid differentiation of human CD34^+^ cells and blocked them at the promyelocytic stage. Morphologically, the promyelocytes had abundant large azurophilic granules and round nuclei with a high nucleocytoplasmic ratio. Some of them had a number of Auer bodies and looked like Faggot cells, which are the typical morphological features of APL cells, and were not seen in the previous murine models (**Figure 2E**). A Southern blot analysis using an EGFP probe revealed that the EGFP^+^/CD45^+^/CD33^+^ cells oligoclonally proliferated *in vivo* (**Figure 2F**).

The induced APL cells were detected in 24 out of the 34 mice (71%) transplanted with *PML-RARA-*transduced CD34 cells (p = 0.0184 in comparison to the control). They were detected in all 16 mice when the calculated number of the transplanted EGFP^+^/CD34^+^ cells per mouse was more than 3,000 (4,655 to 29,728 cells, median: 11,085 cells) (p = 0.0006 in comparison to the control). On the other hand, they were only detected in eight out of 18 mice (44%) transplanted with EGFP^+^/CD34^+^ cells at a density of less than 3,000 (480 to 2,660 cells, median: 1,861 cells) (p = 0.093 in comparison to the control) (**Figure 2G**). This proportion was not dependent on the number of transplanted EGFP^+^/CD34^+^ cells.

These findings demonstrate that a humanized APL model can be successfully established by the transplantation of *PML-RARA*–transduced human CD34 cells into NOG mice.

### Characteristics of the induced APL cells obtained from the humanized *in vivo* model

**Figure 3:**
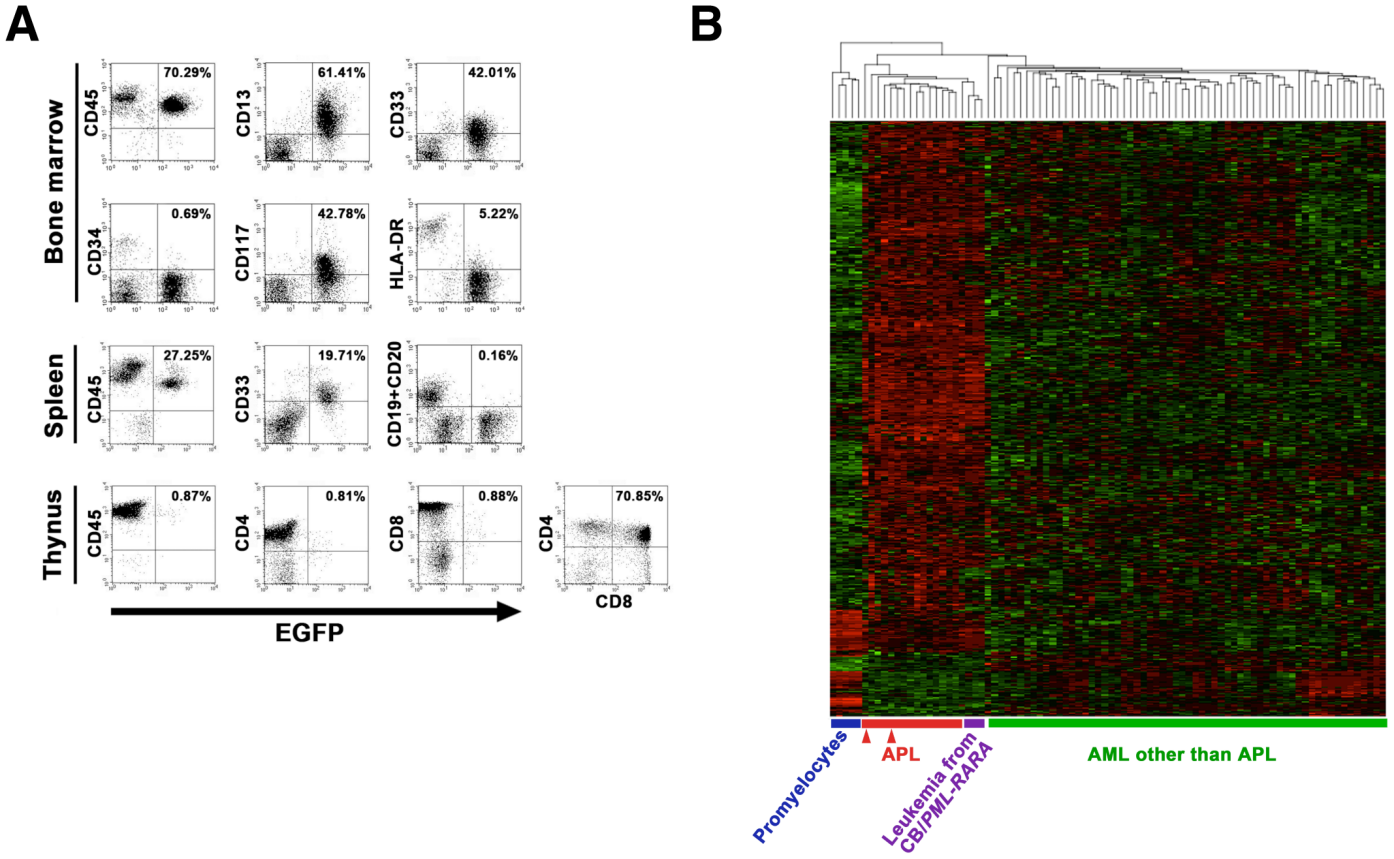
Characteristics of the induced APL *in vivo*. (**A**) The representative expression pattern of cell surface markers in the induced APL cells determined by a flow cytometric analysis. All the scatter plots show the relationships between the EGFP positivity and cell surface marker expression. The whole living cells gated as a propidium iodide-negative fraction in the bone marrow were analyzed. The induced APL cells were recognized as EGFP^+^ cells. A few murine hematopoietic cells, recognized as a human CD45^−^ fraction, were detected in this mouse. (**B**) The heat map of the microarray analysis using the 510 APL-specific genes for the comparison of the induced APL cells (purple, n = 3) with APL (red, n = 16), other types of AML (M0, 1, 2 and 4 in FAB classification, green, n = 62) and normal promyelocytes (blue, n = 5) in a previous study [26]. The red triangles (n = 2) for a total 16 APL cases show the clinical APL samples whose microarray data were obtained in this study.

The induced APL cells were positive for human myeloid markers such as CD13, CD33 and CD117, and were negative for CD34 and HLA-DR, as seen in typical human APL [32]. *PML-RARA* did not contribute to the development of lymphocytes. Human CD19^+^ B-cells in the spleen and human CD4^+^/CD8^+^ T-cells in the thymus were negative for EGFP (**Figure 3A**).

To evaluate the gene expression of the induced APL cells, a microarray analysis was performed, and the expression of the 510 APL-specific genes identified in a previous study was compared with that observed in the clinical AML samples [26]. Two clinical APL samples from our patients were simultaneously evaluated and were aligned in the APL category defined in the study, suggesting that our microarray results were comparable with those in the previous study. The induced APL cells from our models were also classified into the APL category when compared to normal promyelocytes and AML samples other than APL (**Figure 3B**).

In common with this previous study [26], the 3,439 probes (3278 genes) differentially expressed in the induced APL and the AML other than APL ((i) in the Materials and Methods) grouped the induced APL and human primary APL together, separately from the other types of AML (**Figure S4**). The gene sets whose expression in the induced APL cases was not dependent on the myeloid differentiation were also filtered by comparison with the data for normal myeloid cells. They included the genes for immaturity expressed in the induced APL (1,782 probes, including 1,720 genes), the genes upregulated in promyelocyte differentiation not in the induced APL (447 probes, including 427 genes) and the genes specifically induced by *PML-RARA* (466 probes, including 429 genes) ((ii), (iii) and (iv) in the Materials and Methods). Each gene set was analyzed using the DAVID website (david.abcc.ncifcrf.gov/). In the induced APL cases, the genes related to N-Glycan, steroid and heparan sulfate biosynthesis, the spliceosome and pyrimidine metabolism were expressed similar to the levels in normal CD34^+^ cells, and those related to the MAPK signaling pathway were exclusively expressed in comparison to normal myeloid cells, including normal CD34^+^ cells. On the other hand, the genes related to neurotrophin signaling and the cell cycle, as well as those associated with metabolic processes, such as glycolysis/gluconeogenesis, the pentose phosphate pathway and sphingolipid metabolism were downregulated (**Table S2**), thus suggesting that the induced APL exhibited dysregulated signaling and metabolism as differentiated myeloid cells. Additionally, the gene set composed of 573 probes including 547 genes, which fulfilled one or more above criteria (ii) to (iv) in the gene set identified in (i), clearly separated the normal and malignant promyelocytes, such as those of induced and human primary APL (**Figure S5 and Table S3**), as described in the previous study using primary APL cases [26].

To evaluate the additional genetic events that accompanied the integration of *PML-RARA* in the genome, the insertion sites of *PML-RARA* were analyzed. Some of the integration sites of *PML-RARA* were in the introns or exons of genes (**Table S4**). However, they were neither recurrent nor found in the previous whole genome sequence analysis of the APL patients [33]. These findings suggest that *PML-RARA* was a common key event, but that there were various additional genetic events in these induced APL cells, and this finding was compatible with the previous analysis using human primary APL [33].

**Figure 4:**
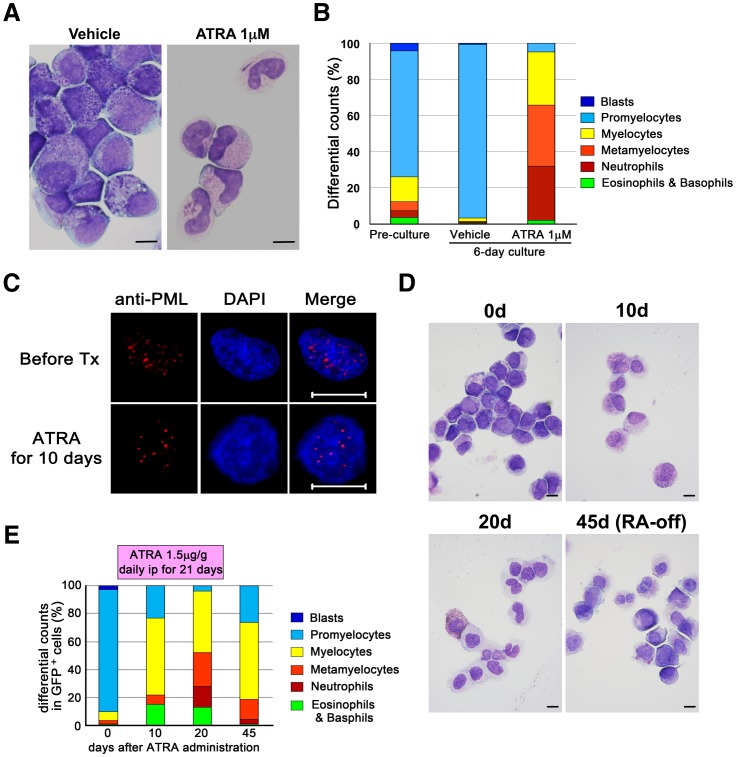
The induction of myeloid differentiation in the APL cells induced by ATRA treatment *in vitro* and *in vivo*. (**A**) Cytospin slides of the induced APL cells cultured with or without 1 µM ATRA for 6 days. The mature neutrophils with Auer rods are seen in the ATRA-treated group. The images were captured with a BX41 microscope (Olympus). The bar indicates 10 µm. (**B**) The differential cellular counts of the induced APL cells cultured with or without ATRA. The average leukocyte differentiation in three independent experiments is shown. (**C**) The results of an immunofluorescent analysis of the PML distribution in the induced APL cells before and after treatment with ATRA. The images were captured with an LSM510 META confocal microscope (Carl Zeiss). All the bars indicate 10 µm. (**D, E**) The induction of myeloid differentiation in the induced APL cells by ATRA *in vivo*. The secondary recipients transplanted with the induced APL cells were then intraperitoneally treated with ATRA for 21 days. Cytospin slides of EGFP^+^/hCD45^+^/hCD33^+^ cells from the secondary recipients transplanted with the induced APL cells are shown (**D**). The images were captured with a BX41 microscope (Olympus). The bar indicates 10 µm. Their differentiated cellular counts were evaluated, and the representative series data are indicated (**E**).

The induced APL cells differentiated into mature neutrophils following treatment with all-*trans* retinoic acid (ATRA) *in vitro* (**Figures 4A and 4B**), which was accompanied by alterations in the PML distribution in the nucleus, from a microspeckled to a speckled pattern (**Figure 4C**). Similarly, ATRA induced transient myeloid differentiation *in vivo* (n = 4), as has been seen in APL patients (**Figures 4D and 4E**).

These findings demonstrate that our induced APL cells recapitulate human APL both phenotypically and functionally.

### Re-transplantable cellular fraction in the induced APL cells

**Figure 5:**
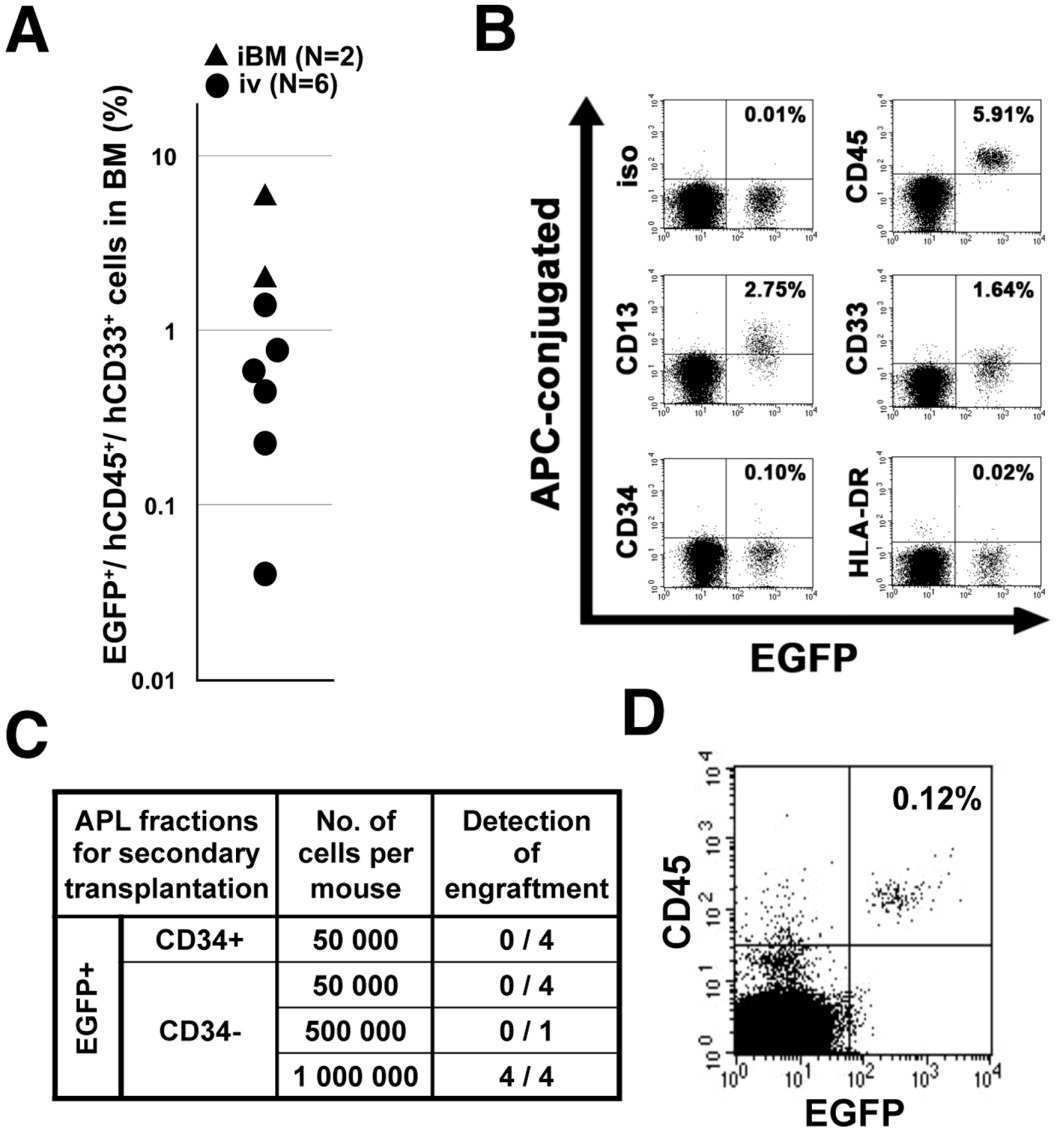
APL-LIC in the humanized APL *in vivo* model. (**A**) The engraftment of the induced APL cells in the secondary recipients. The bone marrow cells were obtained 16 weeks after transplantation and evaluated. Each dot represents a single mouse. (**B**) The immunophenotype of the induced APL cells in the secondary recipients. The representative pattern is shown. (**C**) The engraftment capacity in each fraction from the induced APL cells in the secondary recipients. The bone marrow cells were obtained 16 weeks after transplantation and evaluated. (**D**) The engraftment of the CD34^−^ fraction in the secondary recipients. The engrafted cells are shown as EGFP^+^/CD45^+^ cells.

It is necessary to prove that the resultant induced APL cells possess the reproducibility of APL in the secondary recipients in order to demonstrate their capacity for leukemogenesis. However, previous studies have revealed that primary APL cells exhibit difficultly in engrafting in immunodeficient mice [3], [12]. When the induced APL cells were transplanted into the second recipients, they were proven to be re-transplantable intravenously; 500,000 to 1,000,000 leukemic cells, but not 50,000 cells, were required, and the frequency of APL cells in the secondary recipients was low (0.04% to 1.41%, n = 6). The frequency was still low, even though they were transplanted intramedullary (iBM, 2.00% to 5.98%, n = 2) (**Figure 5A**). The immunophenotype of the engrafted cells was the same as that seen in the primary induced APL cells: they were positive for CD13 and CD33, without the expression of CD34 or HLA-DR (**Figure 5B**), demonstrating that the induced APL retains self-renewal capacity with a low level of transplantation efficiency. In addition, the results suggested that the capacity for engraftment in this xenograft model differed between the CD34^+^ cells transduced with *PML-RARA* and the APL cells mostly composed of the CD34^−^ fraction.

To identify a fraction responsible for the APL maintenance in the induced APL cases, CD34^+^ and CD34^−^ APL fractions were separately collected and transplanted intravenously. The CD34^+^ fraction was pooled because there were very few CD34^+^ cells in each induced APL case (**Figures 3A** **and** **5B**). The CD34^−^ fraction was sorted twice to exclude the CD34^+^ fraction completely. Fifty thousand APL cells in both the CD34^+^ and CD34^−^ fractions failed to engraft in the secondary recipients (0 out of 4 mice in each fraction). Similar to the unsorted cells, one million CD34^−^ fraction cells were able to engraft in recipient mice (4 out of 4 mice) (**Figures 5C and 5D**).

These findings revealed that CD34^−^ induced APL cells exhibit the ability to function as APL-LIC *in vivo*, although the LIC function was not excluded in the CD34^+^ APL fraction.

### The CD34^+^/CD38^+^ progenitors trigger APL by *PML-RARA* induction *in vivo*

**Figure 6:**
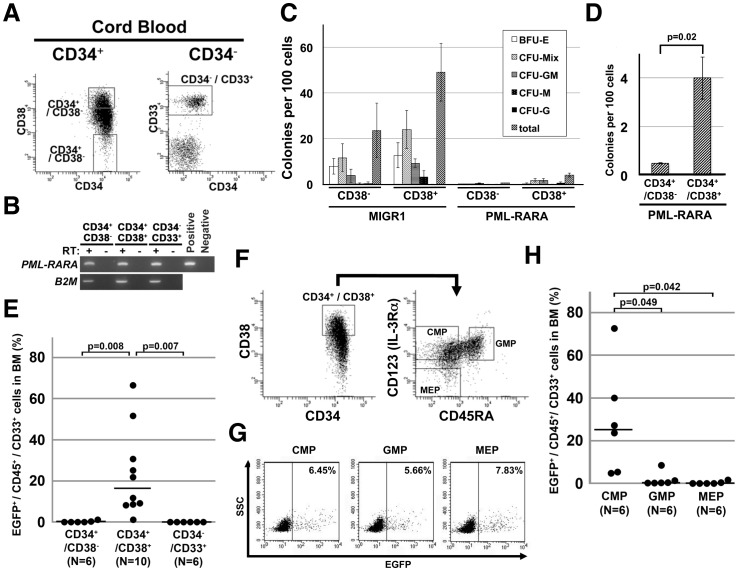
*PML-RARA* targeted human common myeloid progenitors for APL leukemogenesis. (**A**) The sorting strategy for CD34^+^/CD38^−^, CD34^+^/CD38^+^ and CD34^−^/CD33^+^ cells. Human cord blood was first separated into CD34^+^ and CD34^−^ cells by magnetic beads, and then sorted into three fractions by a FACS vantage instrument. (**B**) The expression of *PML-RARA* mRNA in each of the fractions after retroviral transfection. *B2M, beta 2 microglobulin*. The *PML-RARA* expression vector was used as a positive control for the *PML-RARA* analysis. (**C**) A colony-forming assay using *PML-RARA*-transduced CD34^+^/CD38^+^ and CD34^+^/CD38^−^ cells. The average of three independent experiments is shown. The data represent the means ± SD. (**D**) The total numbers of colonies of *PML-RARA*-transduced CD34^+^/CD38^+^ and CD34^+^/CD38^−^ cells shown in (C) are highlighted. The data represent the means ± SD (n = 3). (**E**) The development of the induced APL from CD34^+^/CD38^+^ cells in NOG mice. Each sorted fraction from human cord blood, as seen in (A), was retrovirally transduced with *PML-RARA* and transplanted into irradiated NOG mice. The percentages were determined by the frequency of EGFP^+^/CD45^+^/CD33^+^ cells at 16 to 20 weeks after transplantation. Each dot represents a single mouse. The horizontal line represents the median value. (**F**) The sorting strategy for common myeloid progenitors (CMP), granulocyte-monocytic progenitors (GMP), and megakaryocyte-erythrocyte progenitors (MEP). Human cord blood was separated into CD34^+^ cells by magnetic beads, CD34^+^/CD38^+^ cells were sorted out, and were finally divided into CMP, GMP and MEP by the FACS vantage instrument. (**G**) The transduction efficiency of *PML-RARA* in CMP, GMP and MEP. Representative data are shown. (**H**) The development of the induced APL from the human hematopoietic progenitors in NOG mice. Each sorted progenitor fraction from human cord blood, as seen in (F), was retrovirally transduced with *PML-RARA* and transplanted into irradiated NOG mice. The percentages were determined by the frequency of EGFP^+^/CD45^+^/CD33^+^ cells at 16 to 20 weeks after transplantation. Each dot represents a single mouse. The horizontal line represents the median value.

The findings that the *PML-RARA* transduced-CD34^+^ cells developed APL while the resultant CD34^−^ APL cells exhibited transplantability indicate the possibility that the initiation and maintenance of APL arise at different steps of differentiation, which are not likely to involve the CD34^+^/CD38^−^ fraction, as originally reported in human AML. Therefore, in order to identify a cellular target for *PML-RARA* that effectively develops APL, *PML-RARA* was transduced into fractionated cells: CD34^+^/CD38^−^, CD34^+^/CD38^+^ and CD34^−^/CD33^+^ cells from the cord blood (**Figures 6A and 6B**). The transduction efficiency, as evaluated by EGFP expression, ranged from 1.9% to 5.0% (median: 3.53%, n = 6) in CD34^+^/CD38^−^ cells, 4.5% to 10.6% (median: 10.07%, n = 6) in CD34^+^/CD38^+^ cells and 19.1% to 22.1% (median: 20.63%, n = 4) in CD34^−^/CD33^+^ cells. Because the CD34^+^ fraction from human cord blood possessed a higher proportion of CD34^+^/CD38^+^ (74.5% to 94.2%) than that of CD34^+^/CD38^−^ cells, the presumed absolute number of *PML-RARA* transplanted cells was higher in CD34^+^/CD38^+^ cells than in CD34^+^/CD38^−^ cells (3,430 to 31,800 cells vs 140 to 450 cells per mouse; 22,900 to 27,700 CD34^−^/CD33^+^ cells). One hundred unfractionated human CD34^+^ cells, including both CD34^+^/CD38^−^ and CD34^+^/CD38^+^ cells, were engrafted with multilineage differentiation in our previous study [10], thus suggesting that the transplanted cell numbers were adequate for engraftment in the NOG mice. The induction of *PML-RARA* in CD34^+^/CD38^+^ cells reduced the colony formation capacity and favored the formation of myeloid colonies, as seen in CD34^+^ cells (**Figures 1B and 1C**). On the other hand, the induction of *PML-RARA* in CD34^+^/CD38^−^ cells generated very few colonies in comparison to the MIGR1 control vector-infected CD34^+^/CD38^−^ cells (**Figures 6C and 6D**). Consistent with the results, the induced APL cells were detected mostly in the mice transplanted with CD34^+^/CD38^+^ cells (median, 16.4% in the whole bone marrow cells) (**Figure 6E**). These findings suggest that CD34^+^/CD38^+^ progenitors proliferate and survive more efficiently than CD34^+^/CD38^−^ cells *in vitro* and trigger APL *in vivo* by inducing *PML-RARA*.

### Human common myeloid progenitors develop into APL by inducing *PML-RARA* among CD34^+^/CD38^+^ progenitors

In order to identify the detailed target fraction in CD34^+^/CD38^+^ cells that generates APL with *PML-RARA*, the CD34^+^/CD38^+^ cells were then divided into three fractions based on their expression of CD123 and CD45RA; CMP, GMP and MEP (**Figure 6F**). The retroviral transduction efficiencies of *PML-RARA* into CMP, GMP and MEP were 6.0% to 15.2% (median: 6.6%, n = 9), 3.3% to 8.8% (median: 7.1%, n = 8), and 7.8% to 24.6% (median: 8.5%, n = 7) (**Figure 6G**), and the presumed absolute numbers of *PML-RARA* transduced cells in CMP, GMP and MEP utilized for the transplantation were 5,850 to 15,200, 830 to 4,250, and 1,150 to 2,550 cells per mouse, respectively, which were deduced to directly reflect their proportion in the human cord blood. The frequency of induced APL cells in whole bone marrow cells from transplanted NOG mice was higher when using CMP (median, 25.2%) than GMP and MEP (median, 0.15% and 0.01%, respectively) (**Figure 6H**).

Taken together, these findings obtained using our humanized *in vivo* APL model demonstrate that CMP are a target fraction for *PML-RARA* in the development of APL.

## Discussion

Our present study revealed that a humanized APL model was successfully established by transplantation of human CD34^+^ cord blood transduced with *PML-RARA* into immunodeficient mice. Using this model, we demonstrated that the CMP develop into APL by transducing *PML-RARA* whereas the resultant CD34^−^ APL cells had the ability to maintain the tumor. Our system improves in the following points: The induced APL cells were detected in all of the mice within 150 days if more than 3,000 human CD34^+^ cells infected with *PML-RARA* were transplanted into NOG mice. The resultant leukemia well recapitulated the human disease phenotypically, genetically and functionally, including the presence of Auer rods and Faggot cells, and the expression pattern of cellular surface markers and transcripts, as well as ATRA sensitivity and low leukemia transplantability. These findings demonstrated that this humanized *in vivo* model is suitable for prospectively analyzing the process of APL development in humans.

The cellular subset from which the APL originates is still controversial. Several lines of evidence using *in vivo* experiments have suggested that APL arises in the committed myeloid progenitors, whereas several clinical observations using FISH and RT-PCR analyses suggest that APL arises in earlier progenitors [4]. A recent report using conditional knock-in mice showed that the induction of *PML-RARA* led to dominant proliferation in a stem cell compartment with multilineage potential, but did not result in myeloproliferation, as if the stem cell compartment would not support leukemogenesis in this model [34], [35]. Our *in vitro* and *in vivo* findings are compatible with the previous findings which showed that the generation of *PML-RARA* transgenic mice was only possible by expressing *PML-RARA* in early myeloid cells using the human cathepsin G (hCG) and MRP8 promoters, not the promoters of β-actin, a house-keeping gene, and CD11b which is expressed at a later stage of myeloid differentiation [36].

The leukemogenic function of PML-RARα may require subtle myeloid differentiation, as seen in CMP in the present study; PML-RARα has been reported to possess the inhibitory or toxic effects on the cellular survival, senescence or apoptosis [18], [37], [38]. CMP are still immature enough to easily acquire stemness and are already committed to the myeloid lineage, which may allow *PML-RARA* to dysregulate RARα-dependent myelopoiesis, rather than hematopoietic stem cells, in agreement with the fact that *RARA* is implicated in the regulation of myelopoiesis, including both early stage and terminal differentiation. In this scenario, the expression of *PML-RARA* would induce differentiation of CMP to promyelocytes, but inhibit their terminal differentiation at the same time [19], [39], [40]. Therefore, if the CMP expressing *PML-RARA* acquires stemness, this can result in the development of APL. Our findings showed no engraftment of CD34^+^/CD38^−^ cells with *PML-RARA* transduction *in vivo*, although their leukemogenic activity cannot be denied, as fewer cells were transplanted in comparison to the CD34^+^/CD38^+^ cells. Further analyses are required to evaluate whether CD34^+^/CD38^−^ cells possess the ability to cause APL.

Xenograft models using immunodeficient mice are at present the only method for evaluating the maintenance of human leukemia as LIC. The induced APL generated in the NOG mice had a low engraftment efficiency; however, this biological feature well-reproduced the properties of APL cells found in human patients [3], [12]. It is possible that the difficulty associated with engrafting APL cells, both primary samples and induced APL cells obtained from cord blood, into NOG mice may depend on the different preferences of human hematopoietic cells between humans and mice. The reconstitution of the human hematopoietic system in NOG mice is achieved with the dominant engraftment of B-cells, in comparison to the myeloid lineage cells [10]. This technical feature may affect the engraftment of each of the transplanted cellular subsets in our study. The slow progressive myeloid tumor cells, such as those involved in myelodysplastic syndromes and chronic myeloid leukemia in the chronic phase, were shown to be difficult to engraft in NOD/SCID-β2-microglobulin-deficient or NOG mice (the previous reports [41]–[43] and our unpublished data), suggesting that the engraftment failure did not always indicate a lack of leukemogenecity of the transplanted cells. Therefore, our study confirmed the leukemogenic activity of the CD34^−^ induced APL fraction, although it was not strong. These results are consistent with the previous *in vivo* reports which described the some primary CD34^+^/CD38^+^ and CD34^−^ AML cells could function as LIC *in vivo* [44], [45].

In conclusion, we demonstrated that the induction of *PML-RARA* targeted human CD34^+^ cells, including CMP, and led to their ability to cause APL, and that CD34^−^ APL cells have the capability of maintaining the disease. These findings suggest that it is not necessary that LIC are always consistent with a cellular fraction where leukemia-inducing events occur. Tumor-specific oncogenes, such as *PML-RARA*, effectively function to form tumors with specific characteristics in specific hierarchal stages of myelopoiesis. This model differs from the conventional hierarchal system of AML, in which LIC possess an immature phenotype as seen in hematopoietic stem cells. Since AML is a group of heterogeneous diseases with various causal genetic abnormalities, the present findings will be helpful for the analysis of leukemogenesis in other types of AML which display differentiated leukemic blasts.

## Supporting Information

Figure S1 The detection of *PML-RARA* expression in CD34^+^ cells transduced with *PML-RARA* and their descendent colonies by qualitative RT-PCR. RT, reverse transcription.
(TIF)

Figure S2 Fluorescent images of the colonies derived from the EGFP^+^ and EGFP^−^ fractions of the CD34^+^ cells transduced with *PML-RARA*.
(TIF)

Figure S3 The results of a quantitative analysis of the *PML-RARA* expression in the CD34^+^ cells 48 hours after *PML-RARA* transduction, the differentiated cells from the resultant colonies and the induced APL cells. There were significant differences in the comparison of the expression level of *PML-RARA*. * indicates p values <0.05.
(TIF)

Figure S4 The heat map of the microarray analysis using the 3,439 probes (3,278 genes) that were differentially expressed in the induced APL (n = 3) and the AML other than APL (n = 62). The gene set separated the induced and human primary APL from the cases of AML other than APL. Red triangles (n = 2 from the total of 16 APL cases) show the clinical APL samples whose microarray data were obtained in this study.
(TIF)

Figure S5 The heat map of the microarray analysis performed using the gene set composed of 573 probes (547 genes), which were specifically expressed in the induced APL in comparison to cases of AML other than APL and normal promyelocytes. The gene set clearly clustered the malignant promyelocytes, such as the induced and human primary APL, apart from the normal promyelocytes. Red triangles (n = 2 of a total 16 APL cases) show the clinical APL samples whose microarray data were obtained in this study.
(TIF)

Table S1 The sequences of the PCR primers and probes.
(XLSX)

Table S2 KEGG pathway analysis with the gene sets aberrantly expressed in the induced APL.
(XLSX)

Table S3 The gene set apllied in Figure S5 (573 probes).
(XLSX)

Table S4 The integration sites of PML-RARA in the induced APL cells.
(XLSX)
