# Supplementary figures and images for: Establishment of a Humanized APL Model via the Transplantation of PML-RARA-Transduced Human Common Myeloid Progenitors into Immunodeficient Mice
Source: PLoS One. 2014 Nov 4;9(11):e111082. doi: 10.1371/journal.pone.0111082 (PMC4219701; doi:10.1371/journal.pone.0111082)

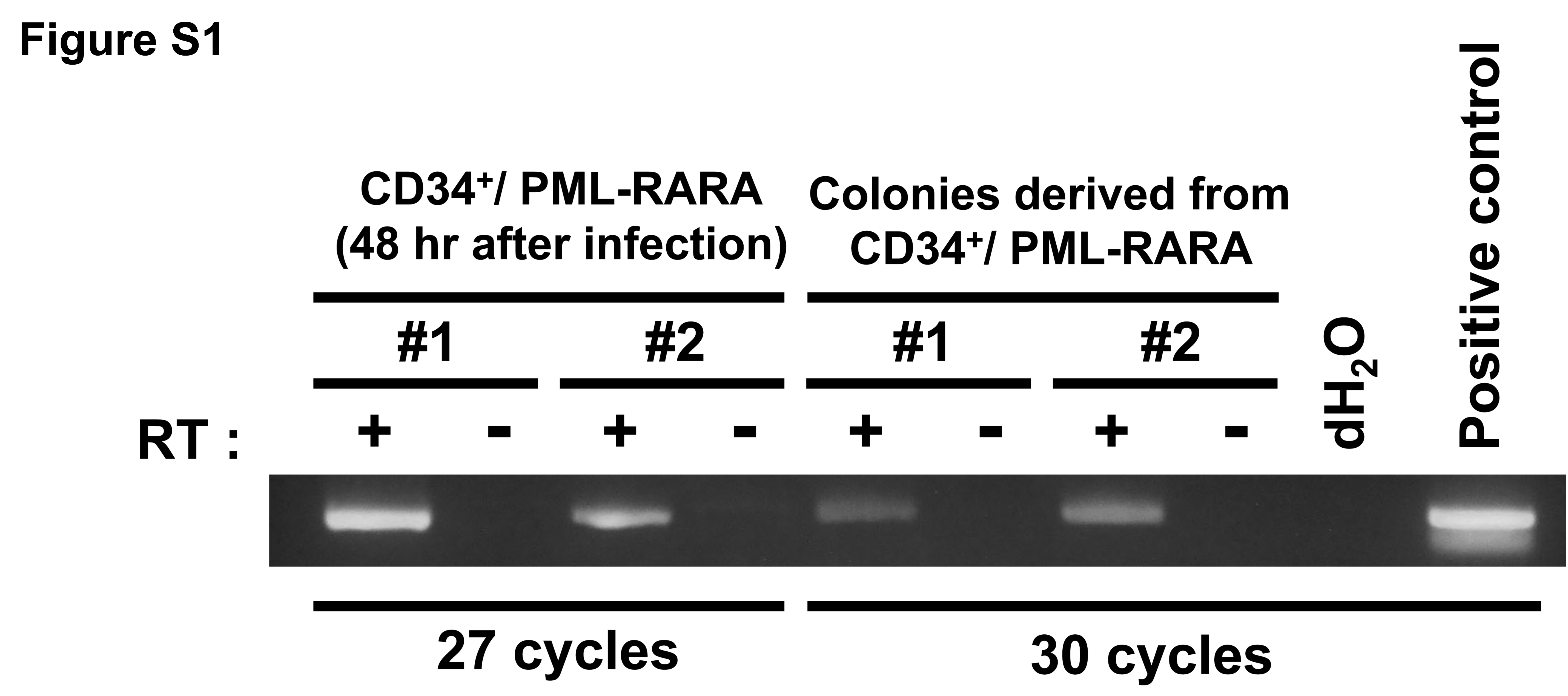

Supplement: Figure S1 — The detection of PML-RARA expression in CD34+ cells transduced with PML-RARA and their descendent colonies by qualitative RT-PCR. RT, reverse transcription. (TIF) [file pone.0111082.s001.tif]

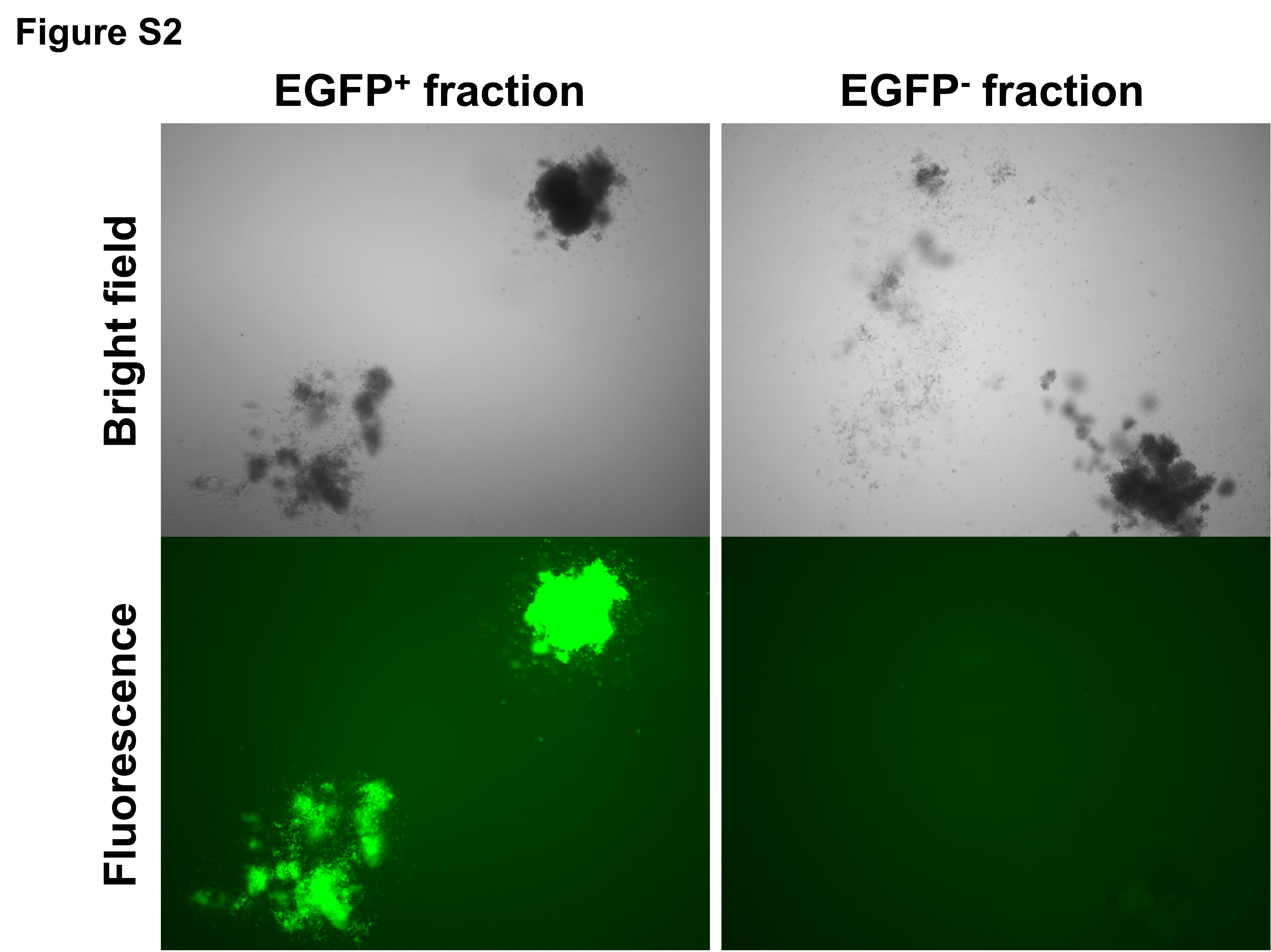

Supplement: Figure S2 — Fluorescent images of the colonies derived from the EGFP+ and EGFP− fractions of the CD34+ cells transduced with PML-RARA. (TIF) [file pone.0111082.s002.tif]

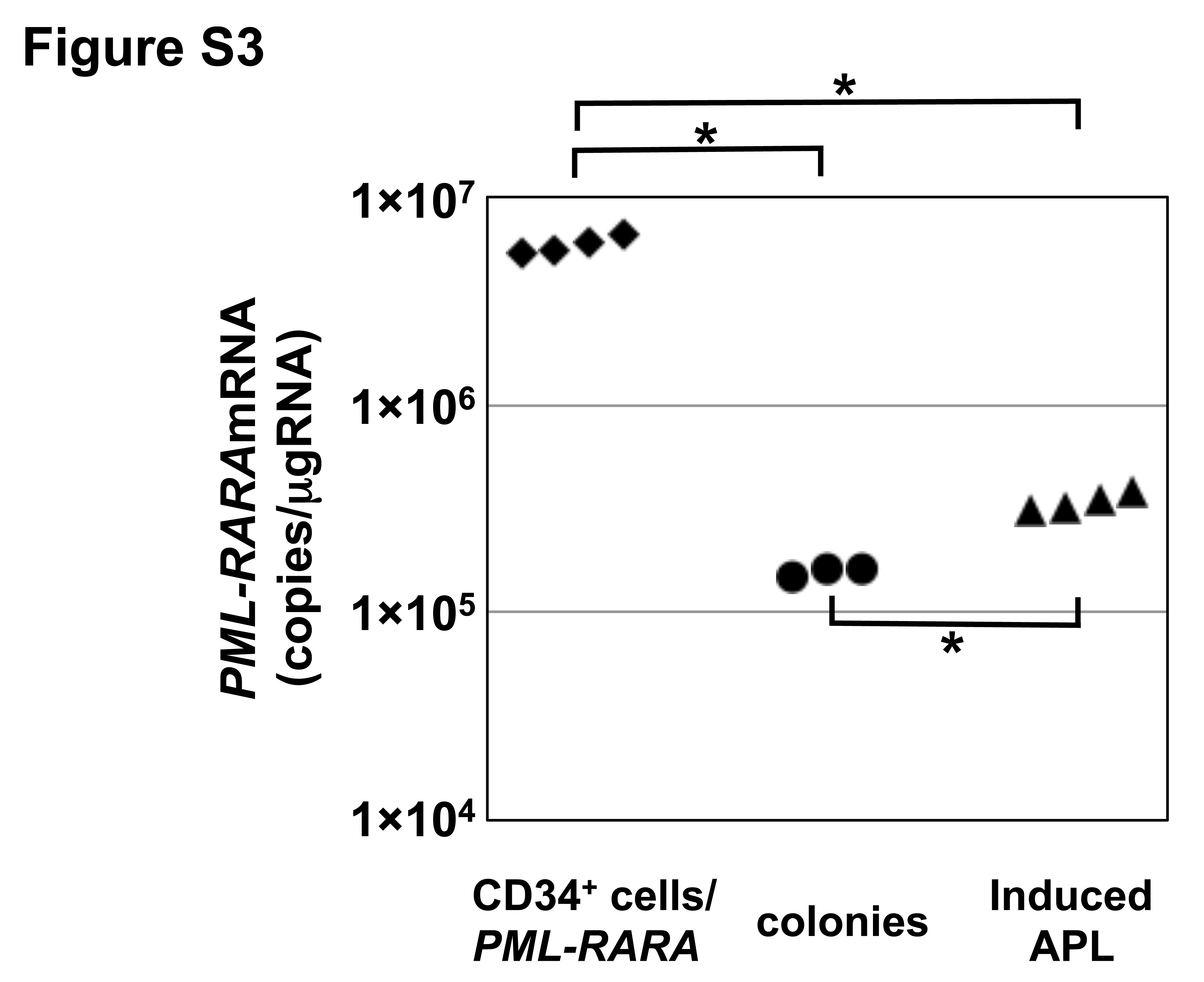

Supplement: Figure S3 — The results of a quantitative analysis of the PML-RARA expression in the CD34+ cells 48 hours after PML-RARA transduction, the differentiated cells from the resultant colonies and the induced APL cells. There were significant differences in the comparison of the expression level of PML-RARA. * indicates p values <0.05. (TIF) [file pone.0111082.s003.tif]

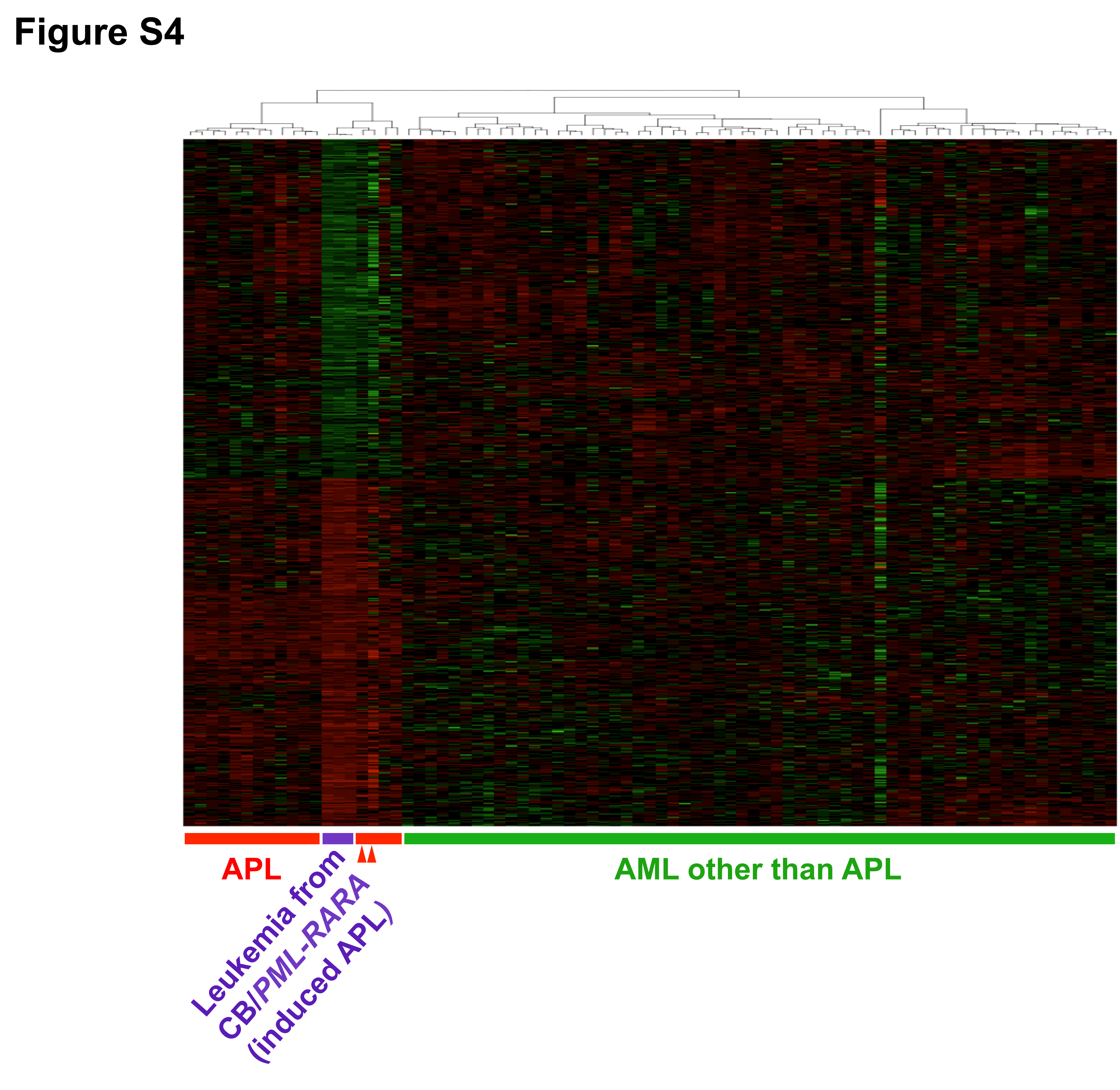

Supplement: Figure S4 — The heat map of the microarray analysis using the 3,439 probes (3,278 genes) that were differentially expressed in the induced APL (n = 3) and the AML other than APL (n = 62). The gene set separated the induced and human primary APL from the cases of AML other than APL. Red triangles (n = 2 from the total of 16 APL cases) show the clinical APL samples whose microarray data were obtained in this study. (TIF) [file pone.0111082.s004.tif]

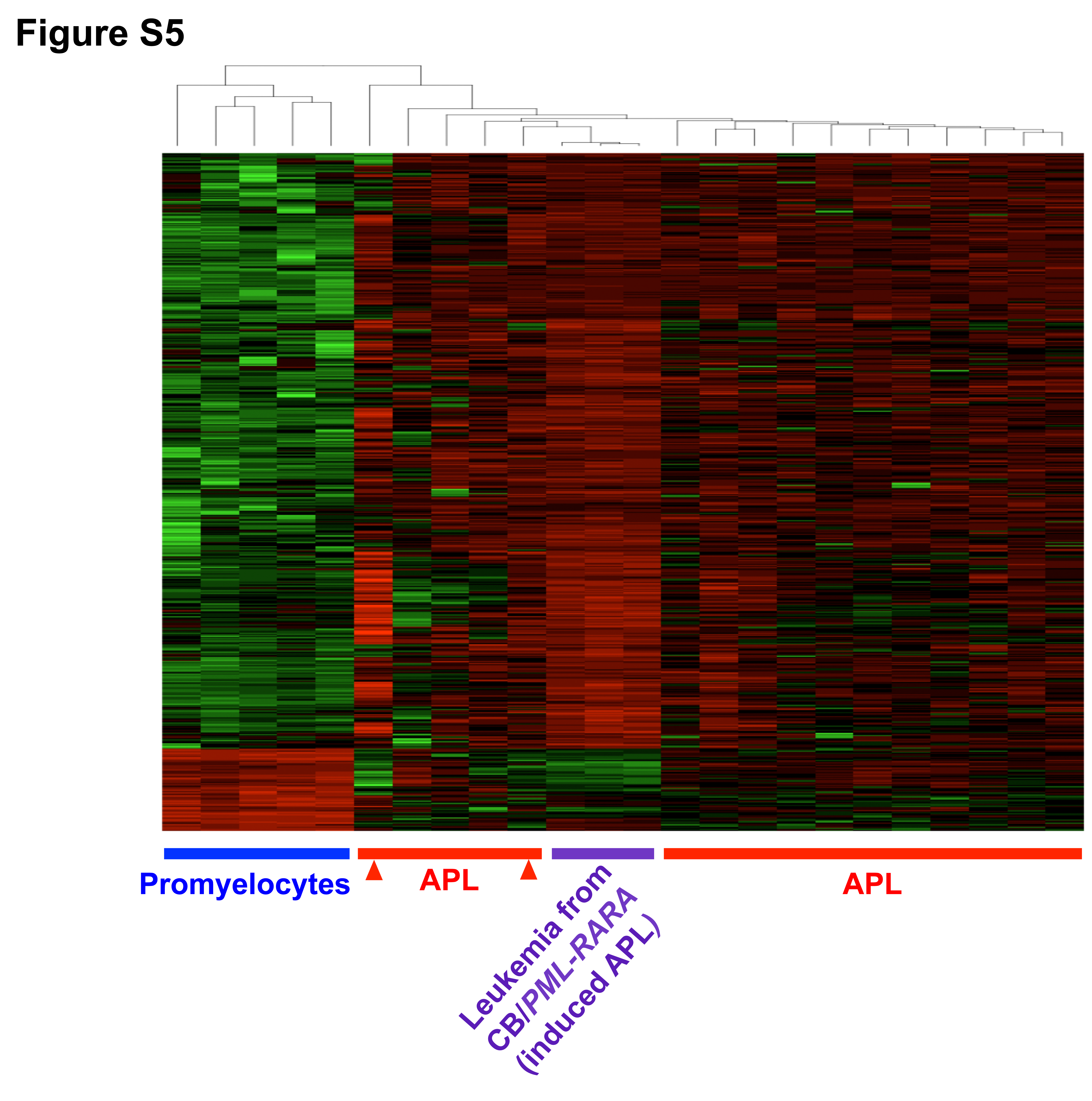

Supplement: Figure S5 — The heat map of the microarray analysis performed using the gene set composed of 573 probes (547 genes), which were specifically expressed in the induced APL in comparison to cases of AML other than APL and normal promyelocytes. The gene set clearly clustered the malignant promyelocytes, such as the induced and human primary APL, apart from the normal promyelocytes. Red triangles (n = 2 of a total 16 APL cases) show the clinical APL samples whose microarray data were obtained in this study. (TIF) [file pone.0111082.s005.tif]
